# Specific mtDNA Mutations in Mouse Carcinoma Cells Suppress Their Tumor Formation via Activation of the Host Innate Immune System

**DOI:** 10.1371/journal.pone.0075981

**Published:** 2013-09-30

**Authors:** Hirotake Imanishi, Gaku Takibuchi, Toshihiko Kobayashi, Kaori Ishikawa, Kazuto Nakada, Masayuki Mori, Yoshiaki Kikkawa, Keizo Takenaga, Noriko Toyama-Sorimachi, Jun-Ichi Hayashi

**Affiliations:** 1 Faculty of Life and Environmental Sciences, University of Tsukuba, Tsukuba, Ibaraki, Japan; 2 Japan Society for the Promotion of Science, Tokyo, Japan; 3 Department of Molecular Immunology and Inflammation, National Center for Global Health and Medicine, Tokyo, Japan; 4 International Institute for Integrative Sleep Medicine, University of Tsukuba, Tsukuba, Ibaraki, Japan; 5 Department of Aging Angiology, Research Center on Aging and Adaptation, Shinshu University School of Medicine, Matsumoto, Nagano, Japan; 6 Mammalian Genetics Project, Tokyo Metropolitan Institute of Medical Science, Tokyo, Japan; 7 Department of Life Science, Shimane University Faculty of Medicine, Izumo, Shimane, Japan; Ohio State University, United States of America

## Abstract

In mammalian species, mitochondrial DNA (mtDNA) with pathogenic mutations that induce mitochondrial respiration defects has been proposed to be involved in tumor phenotypes via induction of enhanced glycolysis under normoxic conditions (the Warburg effects). However, because both nuclear DNA and mtDNA control mitochondrial respiratory function, it is difficult to exclude the possible contribution of nuclear DNA mutations to mitochondrial respiration defects and the resultant expression of tumor phenotypes. Therefore, it is important to generate transmitochondrial cybrids sharing the same nuclear DNA background but carrying mtDNA with and without the mutations by using intercellular mtDNA transfer technology. Our previous studies isolated transmitochondrial cybrids and showed that specific mtDNA mutations enhanced tumor progression as a consequence of overproduction of reactive oxygen species (ROS). This study assessed whether mtDNA mutations inducing ROS overproduction always enhance tumor progression. We introduced mtDNA from senescence-accelerated mice P1 (SAMP1) into C57BL/6J (B6) mice-derived Lewis lung carcinoma P29 cells, and isolated new transmitochondrial cybrids (P29mtSAMP1 cybrids) that overproduced ROS. The inoculation of the cybrids into B6 mice unexpectedly showed that mtDNA from SAMP1 mice conversely induced tumor suppression. Moreover, the tumor suppression of P29mtSAMP1 cybrids in B6 mice occurred as a consequence of innate immune responses of the host B6 mice. Enzyme pretreatment experiments of P29mtSAMP1 cybrids revealed that some peptides encoded by mtDNA and expressed on the cell surface of P29mtSAMP1 cybrids induce increased IL-6 production from innate immune cells (dendritic cells) of B6 mice, and mediate augmented inflammatory responses around the tumor-inoculated environment. These observations indicate presence of a novel role of mtDNA in tumor phenotype, and provide new insights into the fields of mitochondrial tumor biology and tumor immunology.

## Introduction

Mammalian mitochondrial DNA (mtDNA) with pathogenic mutations that induce significant mitochondrial respiration defects causes mitochondrial diseases [1,2]. Moreover, it has been hypothesized that pathogenic mutations in mtDNA may also contribute to aging and age-associated disorders via overproduction of reactive oxygen species (ROS) [1–4]. This hypothesis, the so-called mitochondrial theory of aging, is partly supported by studies in mtDNA mutator mice, which possess a nuclear-encoded mtDNA polymerase with a defective proofreading function, resulting in enhanced expression of respiration defects with age and the subsequent expression of premature aging phenotypes [5–7]. Furthermore, the accumulation of pathogenic mtDNA mutations with age and the resultant mitochondrial respiration defects also have been proposed to be involved in the tumor development, given that these defects induce enhanced glycolysis under normoxic conditions (i.e., the Warburg effect), thus providing a survival advantage for tumor cells even under hypoxic conditions [1–4].

However, there is as yet no convincing evidence for the contribution of mtDNA mutations to aging and tumor phenotypes because of the dual control of mitochondrial respiratory function by both nuclear DNA and mtDNA [1,2] and the resultant difficulty of excluding the possible involvement of nuclear DNA mutations in the expression of these phenotypes. Therefore, to examine the contribution of mtDNA mutations to aging or tumor phenotypes, it is very important to use intercellular mtDNA transfer technology to generate transmitochondrial cybrids or transmitochondrial mito-mice that share the same nuclear DNA background but carry mtDNA with and without the mutations. In our previous studies, we generated such transmitochondrial cybrids [8,9] and transmitochondrial mito-mice [10,11] and showed that specific mtDNA mutations regulated tumor progression as a consequence of their induction of ROS overproduction while they did not regulate aging phenotypes [11].

The current study addressed the issue of whether mtDNA mutations inducing ROS overproduction always enhance tumor progression. For this, we isolated new transmitochondrial P29mtSAMP1 cybrids that carry mtDNA from senescence-accelerated mice P1 (SAMP1) [12,13] and express ROS overproduction, and examined their tumor phenotypes. The results were unexpected, since tumor phenotypes of Lewis lung carcinoma P29 cells derived from C57BL/6J (B6) strain mice were suppressed by the introduction of mtDNA from SAMP1 mice. These observations suggest that some of mtDNA mutations can function as suppressors of tumor phenotypes rather than enhancers.

## Materials and Methods

### Cell lines and cell culture

The P29 cells, originated from B6 mouse–derived Lewis lung carcinoma cells, were established in our previous study [14]. The mtDNA-less P29 cells (ρ^0^ P29 cells), and the transmitochondrial cybrids were grown in DMEM (Sigma, St. Louis, MO, USA) containing 10% fetal calf serum (Sanko Junyaku, Tokyo, Japan), 50 mg/ml uridine (Sigma, St. Louis, MO, USA), and 0.1 mg/ml sodium pyruvate (Wako Pure Chemical Industries, Osaka, Japan).

### Isolation of transmitochondrial cybrids

We used ρ^0^ P29 cells as nuclear donors and mtDNA recipients for isolation of the transmitochondrial cybrids (P29mtSAMP1 cybrids). As mtDNA donors, we used platelets from SAMP1 mice. Platelet-containing supernatants were prepared by low-speed centrifugation (70 × *g* for 15 min) of blood obtained from SAMP1 mice. Platelets were fused with ρ^0^ P29 cells by polyethylene glycol. Selective isolation of P29mtSAMP1 cybrids was attained in the selection medium without uridine and sodium pyruvate (UP^-^), so that unfused nuclear donor ρ^0^ P29 cells could be excluded by the selection.

### Mice

Mice of the inbred strain B6 were obtained from CLEA (Tokyo, Japan). The immunodeﬁcient B6 *Rag2* ^*-/-*^ mice were obtained from Taconic (Hudson, NY, USA). The knockout mice of B6 *Myd88/Trif*, *TLR-3*, *TLR-7*, and *TLR-9* were obtained from Bioindustry Division of Oriental Yeast (Tokyo, Japan). B6 CD11c-DTR mice, which loose CD11c^+^ dendritic cells (DCs) by administration of diphtheria toxin (DTx) due to expression of its receptor (DTR) exclusively in CD11c^+^ DCs [15], were obtained from Dr. S. Koyasu (Keio University) with permission of Dr. D. R. Littman (New York Univ. Sch. Med.). To obtain B6 NK^Red^ mice, B6 mice received continuous intraperitoneal injection of anti-NK1.1 antibody (BioLegend, San Diego, CA, USA) from 1 week after the birth (10 µg/week for 1-week–old mice, 20 µg/week for 2-week–old mice, 50 µg/week for 3- and 4-week–old mice, and 100 µg/week for >5-week-old mice). All the immunodeﬁcient mice used here share a B6 nuclear DNA background. SAMP1 males were obtained from Japan SLC (Shizuoka, Japan). Animal experiments were performed in accordance with protocols approved by the Experimental Animal Committee of the University of Tsukuba, Japan (Approval number: 12070), and Institutional Animal Care and Use Committee of the Research Institute National Center for Global Health and Medicine (Approval number: 12023).

### Genotyping of mtDNAs in the cybrids

To confirm transfer of mtDNAs from SAMP1 mice into P29mtSAMP1 cybrids, restriction enzyme digestion of the PCR products was carried out. For recognition of an A11181G mutation in the *ND4* gene of SAMP1 mice (Table 1), a 194-bp fragment containing the 11181 site was amplified by PCR. The sequences from nucleotide position 11,102 to 11,125 (AAC AAT ACT AAT AAT CGC ACA TGG) and nucleotide position 11,295 to 11,272 (CTA TTA GAT TGA TTG AAG GGG GTA) were used as oligonucleotide primers. Combination of the PCR-generated mutation with the A11181G mutation creates a restriction site for *Eag*I, and generates 115-bp and 79-bp fragments on *Eag*I digestion. The restriction fragments were separated in 3% agarose gel.

**Table 1:** Comparison of sequences of mtDNA from transmitochondrial cybrids.

**Gene**	**Position**	**B6**	**P29mtSAMP1**	**P29mtC3H**	**Amino acid change**
***16SrRNA***	2256	T	C	–	–
***ND2***	4794	C	–	T	T294I
***COX3***	9348	G	–	A	V248I
***ND3***	9461	T	C	C	Silent
***ND4***	11181	A	G	–	T337A
***ND5***	12048	T	–	C	F103L
	12395	C	A	–	I218M
	13052	T	C	–	Silent
**GenBank accession no.**		AY172335	AP013054	AP013031	

### Estimation of the ROS levels

ROS generation was detected with 2′-,7′-dichlorofluorescein diacetate (DCFH-DA) (Invitrogen, Carlsbad, CA, USA). Cells were incubated with 5 µM DCFH-DA for 10 min at 37°C in serum-free DMEM, washed twice with Dulbecco’s phosphate-buffered saline (DPBS), and then immediately analyzed with a FACScan flow cytometer (Becton Dickinson, Mountain View, CA, USA).

### Assays of tumor phenotypes

For testing tumor formation phenotype and metastatic potential, 5 × 10^6^ cells in 100 µl PBS were injected subcutaneously into the back of 6-week–old male B6 mice. Tumor growth was monitored assuming spherical growth of tumors. When a tumor mass was visually detectable, its maximum (a) and minimum (b) diameters and height (h) were recorded twice per week. The volume of each tumor (V) was calculated according to the following formula: V =πabh/6. The recipient mice were sacrificed by cervical dislocation when the tumor volume reached 500 mm^3^.

### Cytotoxic assay

^51^Cr-release assay was performed to examine cytotoxic activity of cells. For preparation of poly (I:C)-activated natural killer (NK) cells, 100 µg of poly (I:C) (Sigma, St. Louis, MO, USA) was intraperitoneally injected to B6 *Rag2* ^*-/-*^ mice, and spleen cells were isolated as effector cells 18 h after the injection. The target cells (P29mtB6 and P29mtSAMP1 cybrids) were labelled with ^51^Cr (3700 KBq/10^6^ cells) for 30 min at 37°C and used as target cells in the killer assay. After removing the excess ^51^Cr by washing cells with the medium, the target cells were plated in V-bottom 96-well plates at 10^4^ cells/well and mixed with effector cells in a final volume of 200 µl, and the cytotoxic assay was performed. Spontaneous release values were obtained from target cells in medium alone, whereas total release values were obtained from target cells lysed in 1% Nonidet P-40 in distilled water. The percentage of specific lysis was calculated as follows: % of killing = [(the mean cpm released in the presence of effector cells - spontaneous release cpm)/(total release cpm - spontaneous release cpm)] × 100. Spontaneous release values of target cells were less than 20% of total release values in all experiments.

### Estimation of the cytokines produced by DCs

Bone marrow (BM) cells were isolated by flushing femurs and tibiae of euthanized mice with RPMI 1640 medium supplemented with 5% heat-inactivated FCS. The BM cells were treated with a Tris-ammonium chloride buffer to lyse red blood cells and plated at a concentration of 10^6^ cells/ml into a six-well culture dish in culture medium consisting of RPMI 1640 medium supplemented with 10% FCS, 10 mM HEPES, 2 mM l-glutamine, 1 mM sodium pyruvate, 50 µM 2-mercaptoethanol, 1% (v/v) nonessential amino acids, 100 U/ml penicillin, 100 µg/ml streptomycin, and 100 ng/ml murine Flt3L. Every 4 days of culture, half of the medium was removed and fresh cytokine-supplemented culture medium was added back into the cultures. After 8 days, DCs were harvested, plated into 96-well plates mixed with P29mtB6 or P29mtSAMP1 cybrids. After 48 h incubation, the supernatant was harvested to measure cytokine production by DCs by using an ELISA kit (BioLegend, San Diego, CA, USA) and Mouse Inflammation CBA Kit (Becton Dickinson, Mountain View, CA, USA).

### Statistical analysis

All analyses and experiments were conducted at least three times. Differences between groups of values were assessed by using a 2-tailed unpaired Student’s *t*-test. All values are means ±S.D. *P* values < 0.05 were considered to be statistically significant.

## Results

### Isolation and characterization of transmitochondrial P29mtSAMP1 cybrids

**Figure 1:**
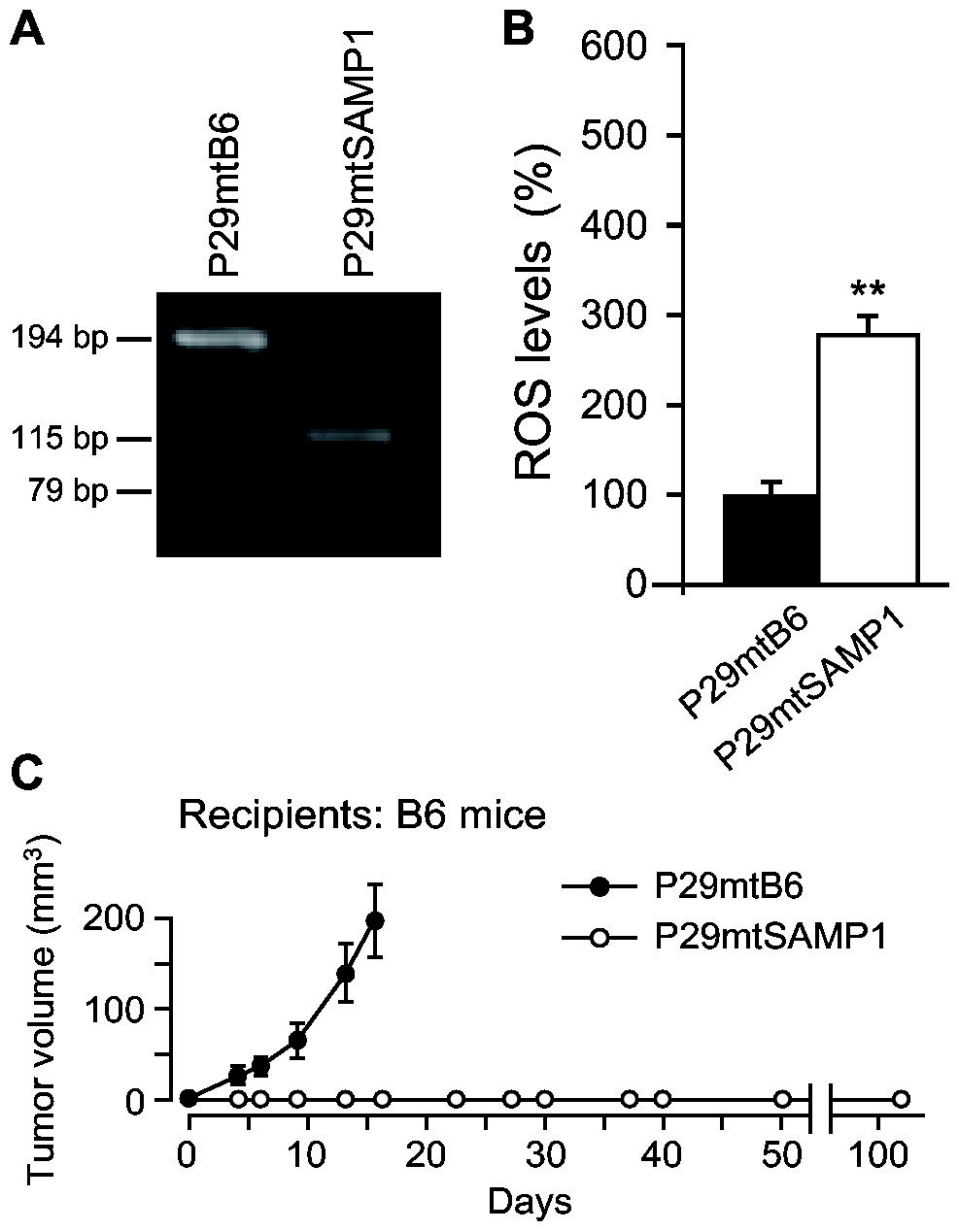
Characterization of transmitochondrial P29mtSAMP1 cybrids. (**A**) Genotyping of mtDNA in P29mtSAMP1 cybrids. As control cybrids, we used P29mtB6 cybrids with mtDNA from B6 mice [16]. To identify the A11181G mutation in the mtDNA of P29mtSAMP1 cybrids (Table 1), the PCR products were digested with the restriction enzyme *Eag*I. mtDNA with the A11181G mutation from SAMP1 mice in P29mtSAMP1 cybrids produced 115-bp and 79-bp fragments due to the gain of an *Eag*I site through an A to G substitution at the nucleotide position 11181, whereas B6 mtDNA without the mutation in P29mtB6 cybrids produced a 194-bp fragment. (**B**) Effects of mtDNA transfer from SAMP1 mice on the ROS levels of P29mtSAMP1 cybrids. P29mtB6 and P29mtSAMP1 cybrids (1 × 10^6^ cells) treated with 5 µM DCFH-DA underwent flow cytometric analysis for quantitative estimation of ROS (H_2_O_2_). (**C**) Tumor phenotypes of P29mtSAMP1 cybrids. For examination of tumor phenotypes (tumorigenicity and spontaneous metastasis), 5 × 10^6^ cybrids were inoculated subcutaneously into B6 mice. P29mtSAMP1 cybrids did not form primary tumor masses, whereas P29mtB6 cybrids expressed tumorigenicity by forming primary tumor masses within 10 days after inoculation. Neither types of cybrids formed lung nodules, suggesting that neither had a high potential for metastasis. **P < 0.01.

According to the mitochondrial theory of aging [1–4], accumulation of mtDNA mutations with age and the resultant overexpression of ROS are responsible for aging phenotypes. Given that SAMP1 mice express accelerated aging phenotypes [12,13], their mtDNA can be expected to induce ROS overproduction. Therefore, we transferred mtDNA from SAMP1 mice into mtDNA-less Lewis lung carcinoma P29 cells (ρ^0^ P29 cells) derived from B6 strain mice, and isolated transmitochondrial P29mtSAMP1 cybrids carrying mtDNA from SAMP1 mice (Table S1). Transfer of mtDNA from SAMP1 mice into P29mtSAMP1 cybrids was confirmed by restriction enzyme digestion of PCR products amplified by using mismatched primers (Figure 1A). As control cybrids, we used P29mtB6 cybrids with mtDNA from B6 strain mice, which we isolated in our previous study and which formed primary tumors in syngenic B6 mice after subcutaneous inoculation [16]. Thus, both P29mtB6 and P29mtSAMP1 cybrids share the nuclear DNA from B6 mice but carry mtDNA from B6 mice and from SAMP1 mice, respectively.

Because P29mtSAMP1 cybrids demonstrated ROS overproduction (Figure 1B), we then examined whether mtDNA mutations that induce ROS overproduction always enhance tumor progression (metastasis), as we proposed in our previous study [8]. For examination of spontaneous metastasis, we inoculated 5 × 10^6^ cybrids subcutaneously into B6 mice. However, we obtained the unexpected results that P29mtSAMP1 cybrids did not form primary tumor masses even after 100 days, whereas P29mtB6 cybrids with mtDNA from B6 mice formed tumor masses within 10 days after inoculation (Figure 1C). Therefore, mutations in mtDNA from SAMP1 mice might be responsible for the suppression of the tumor formation phenotype of Lewis lung carcinoma P29 cells. Taken together with our previous observation [9], these results suggest that some mtDNA mutations function as tumor suppressors (Figure 1C), while the other mtDNA mutations function as tumor enhancers [9], even though they both induce ROS overproduction.

### Involvement of the immune system in the suppression of P29mtSAMP1 tumor formation

The above result that tumor forming ability of P29 Lewis lung carcinoma was suppressed by introducing SAMP1 mtDNA mutations into P29 cells implied a crucial role of mtDNA mutations in tumor suppression. We previously found that transmitochondrial cybrids with nuclear DNA from P29 cells and mtDNA from NZB strain mice (P29mtNZB cybrids) did not form tumor masses in B6 mice due to the recognition and elimination of these cybrids by the innate immune system of B6 mice, although P29mtB6 cybrids form tumor masses under the same host environment [16]. NZB mtDNA possesses a number of non-pathogenic, polymorphic alterations of nucleotides compared with B6 mtDNA, and P29mtNZB cybrids appeared to be recognized and eliminated by the innate immune system just like allogenic (non-self) graft in the host B6 mice [16].

**Figure 2:**
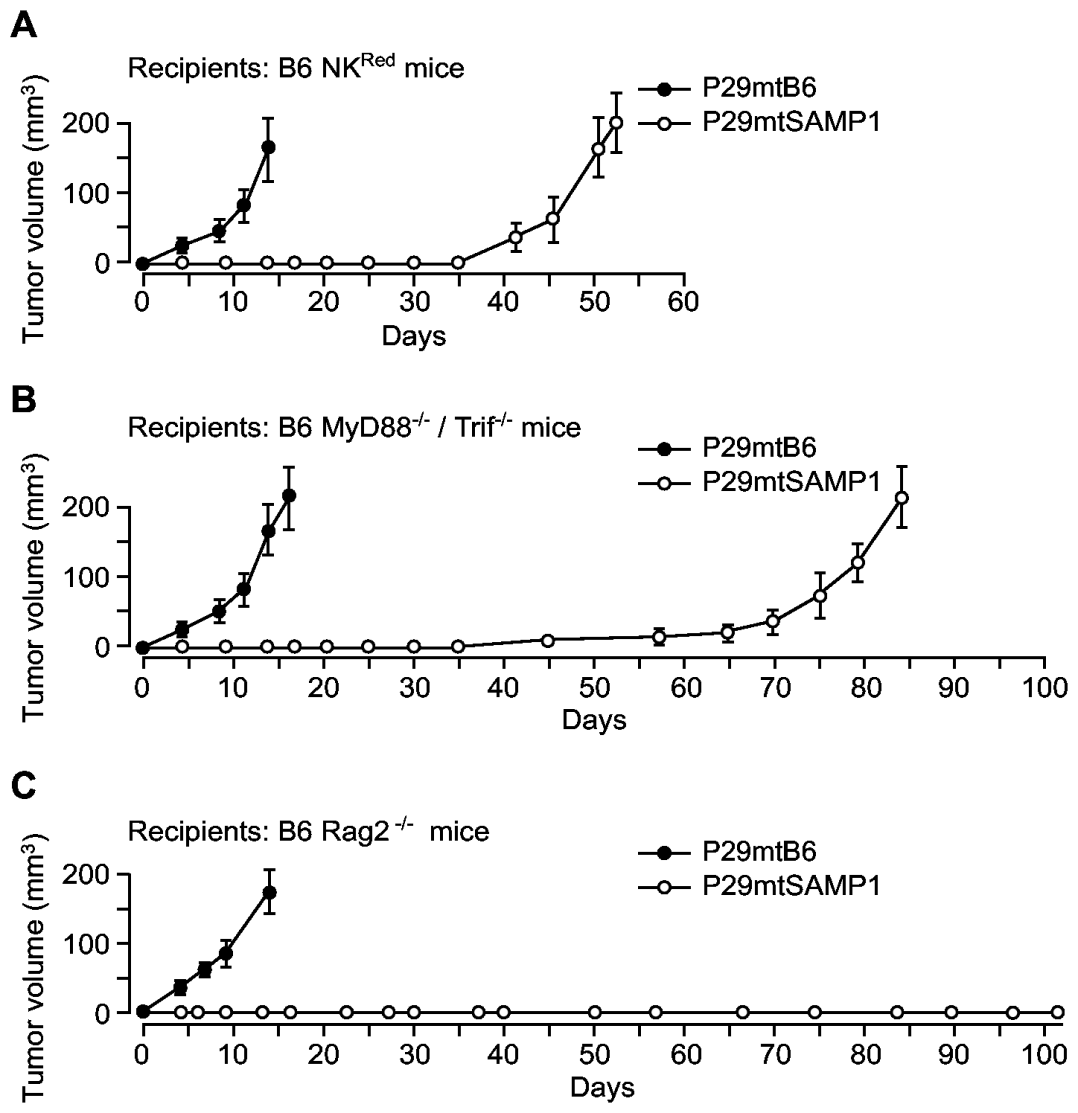
Involvement of immune systems of B6 mice in the suppression of tumor formation of P29mtSAMP1 cybrids. P29mtB6 and P29mtSAMP1 cybrids were inoculated under the skin of (**A**) B6 NK^Red^ mice, which lack NK cells, (**B**) B6 *Myd88* ^*-/-*^ *Trif* ^*-/-*^ mice, which lack a functional innate immune system, and (**C**) B6 *Rag2* ^*-/-*^ mice lacking the acquired immune system (*n* = 3 per group). P29mtSAMP1 cybrids formed primary tumor masses in B6 mice only in the absence of functional innate immune system, indicating that innate immune system is responsible for suppression of the tumor formation phenotype of P29mtSAMP1 cybrids.

We therefore examined whether suppression of tumor formation by P29mtSAMP1 cybrids is due to the immune responses of host B6 mice (Figure 2). To this end, we used B6-background immune deficient mice including NK^Red^ mice, which have a reduced number of NK cells as the result of intraperitoneal injection of anti-NK1.1 antibody, and B6 *MyD88* ^*-/-*^ */Trif* ^*-/-*^ mice, which are deficient in the innate immune system due to the functional impairment of Toll-like receptors and some cytokine receptors such as IL-1R and IL-18R. The P29mtSAMP1 cybrids were able to form tumors in both B6 NK^Red^ mice (Figure 2A) and B6 *MyD88* ^*-/-*^ */Trif* ^*-/-*^ mice (Figure 2B), although they showed longer latent periods than those of P29mtB6 cybrids to form measurable tumor volumes. Probably, the incomplete deficiency of innate immune system in these mice may be responsible for the partial suppression of tumor formation. These results indicated that suppression of tumor formation of P29mtSAMP1 cybrids is the result of host immune responses, and that the innate immune cells including NK and other immune cells that utilize Myd88 or Trif adaptor molecules for their function, are involved in the suppression of tumor formation of P29mtSAMP1 cybrids in the B6 mice.

We further evaluated the contribution of the acquired immune system to tumor suppression of P29mtSAMP1 cybrids using B6 *Rag2* ^*-/-*^ (T and B cell–deficient) mice, which have a defective acquired immune system due to deficiency of the recombination-activating gene. P29mtSAMP1 cybrids failed to form tumors in B6 *Rag2* ^*-/-*^ mice (Figure 2C). These results indicate that the acquired immune system of host B6 mice was not involved in the suppression of tumor formation of P29mtSAMP1 cybrids (Figure 1C).

### Involvement of NK cells and DCs in tumor suppression of P29mtSAMP1 cybrids

**Figure 3:**
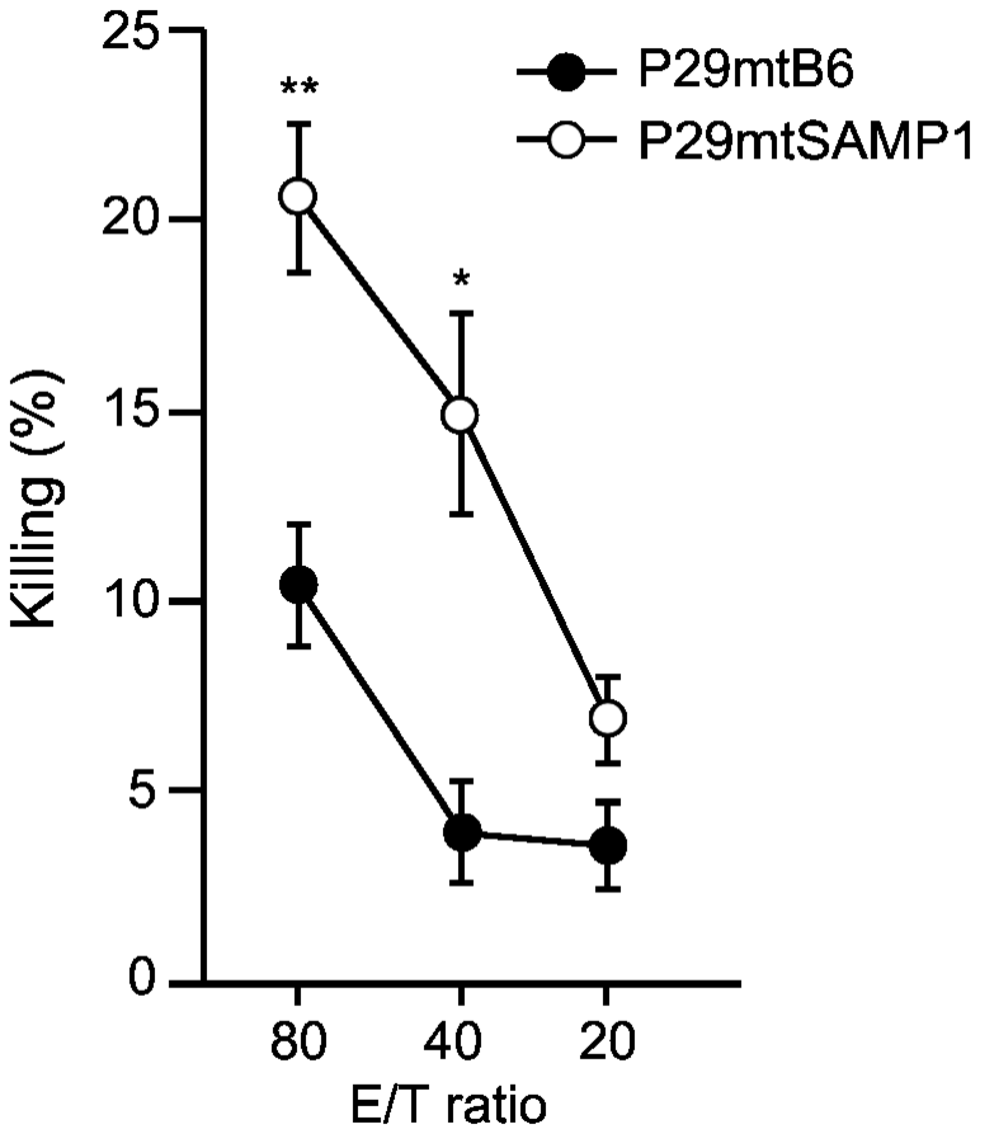
Involvement of NK cells in the specific killing of P29mtSAMP1 cybrids. Cytotoxic assays of P29mtB6 and P29mtSAMP1 cybrids (target cells) used B6 *Rag2* ^*-/-*^ splenocytes (effector cells) without T cells and B cells at indicated E/T ratios (effector cells to target cells ratios). Specific killing of P29mtSAMP1 cybrids was observed. *P < 0.05, **P < 0.01.

The tumor formation in NK^Red^ mice suggested that the NK cells of B6 mice are involved in the suppression of tumor formation of P29mtSAMP1 cybrids (Figure 2A). To further provide evidence for the contribution of NK cells in tumor suppression of P29mtSAMP1 cybrids, we performed *in vitro* cytotoxic assays by co-cultivation of ^51^Cr-incorporated cybrids with activated NK cells prepared from the B6 *Rag2* ^*-/-*^ mice. The results showed that activated NK cells were more cytotoxic to P29mtSAMP1 cybrids than to P29mtB6 cybrids (Figure 3). Thus, activated NK cells could recognize and kill P29mtSAMP1 cybrids preferentially.

**Figure 4:**
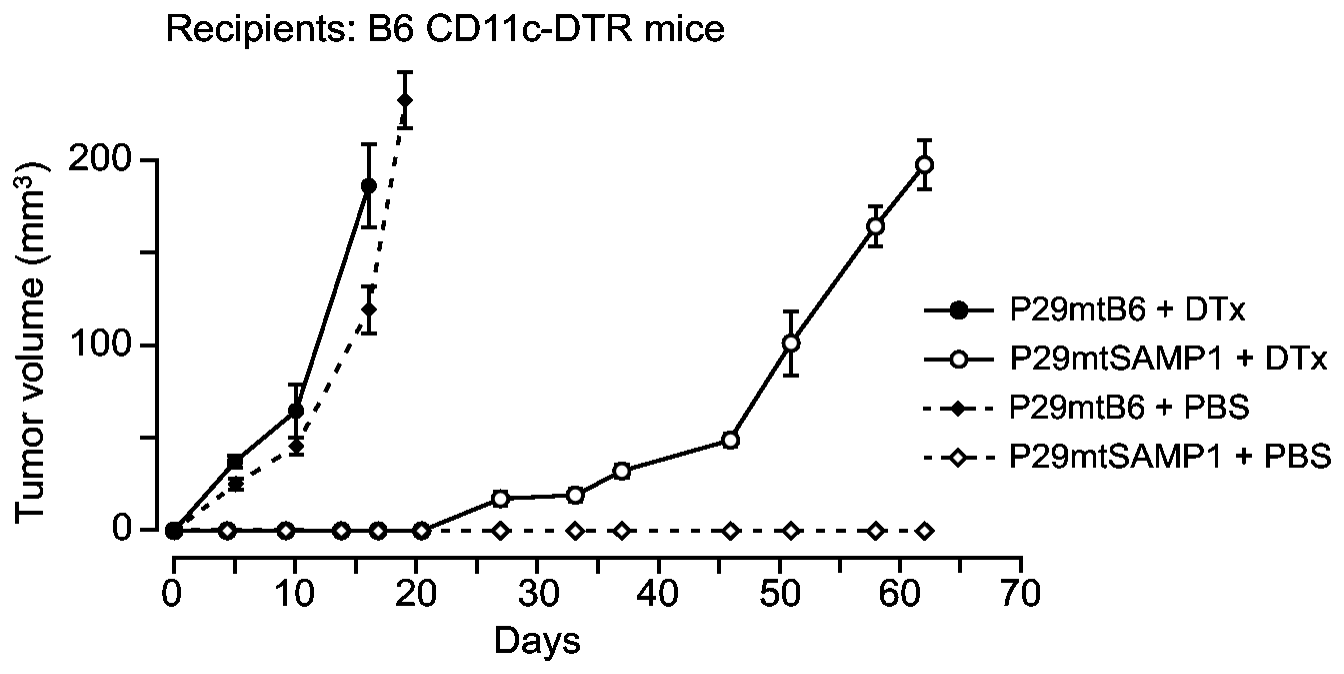
Involvement of DCs in the suppression of tumor formation of P29mtSAMP1 cybrids in B6 mice. P29mtB6 and P29mtSAMP1 cybrids were inoculated under the skin of B6 CD11c-DTR mice, which have lost DCs by the administration of DTx (*n* = 3 per group). P29mtSAMP1 cybrids formed primary tumor masses in B6 CD11c-DTR mice, only when the mice were treated with DTx, indicating that DCs are at least in part involved in suppression of the tumor formation phenotype of P29mtSAMP1 cybrids.

The experiments using B6 *MyD88* ^*-/-*^ */Trif* ^*-/-*^ mice suggested that in addition to NK cells, other innate immune cells, such as DCs, are involved in the suppression of tumor formation of P29mtSAMP1 cybrids (Figure 2B). Therefore, we examined the contribution of DCs by using B6 CD11c-DTR mice, which lack DCs after the administration of DTx owing to exclusive expression of its receptor (DTR) in CD11c+ DCs. Depletion of DCs by DTx allowed tumor formation of P29mtSAMP1 cybrids, although they showed longer latent periods than those of P29mtB6 cybrids (Figure 4).

**Figure 5:**
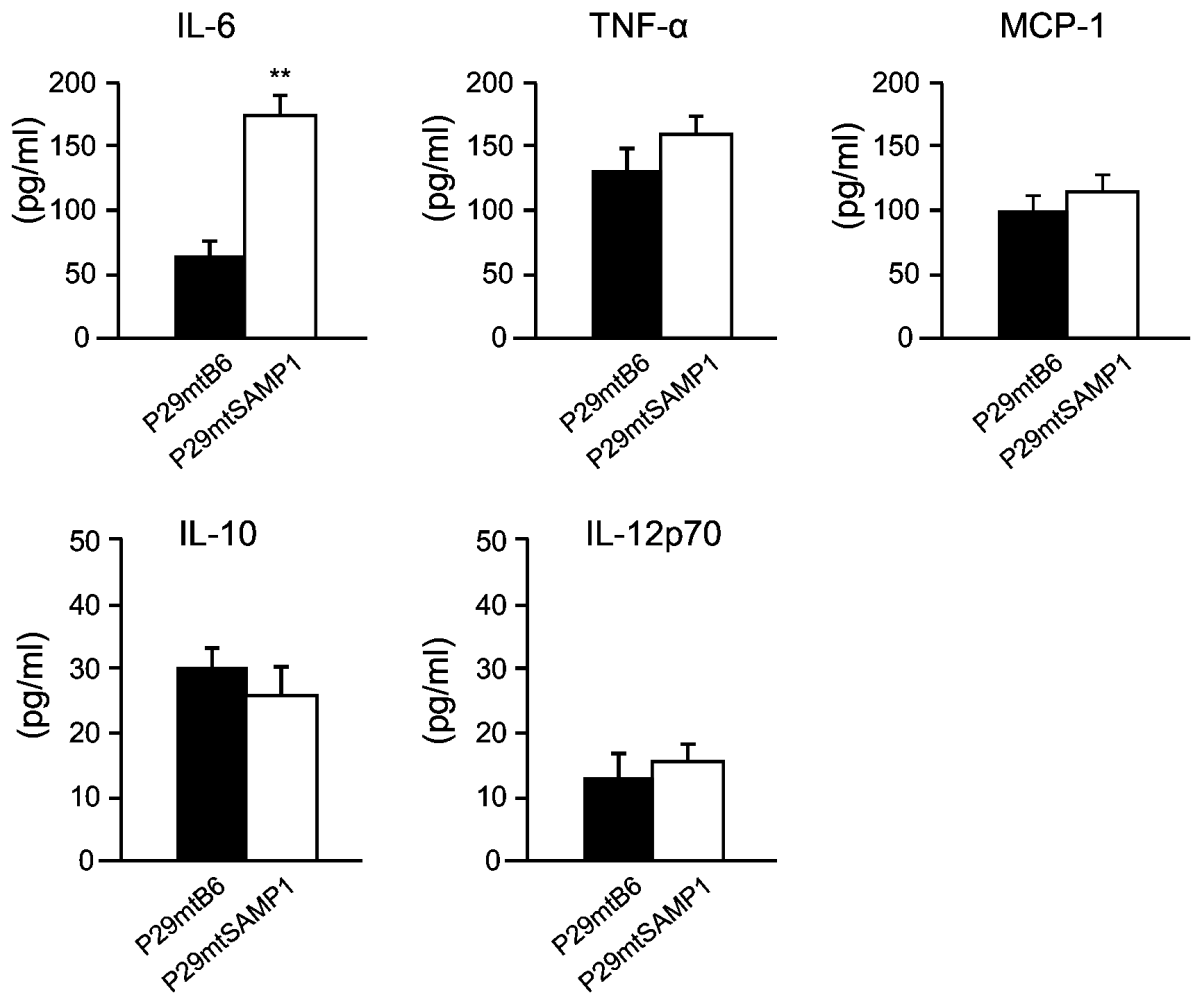
Identification of the cytokines secreted from DCs and increased in amount after co-culture with P29mtSAMP1 cybrids. DCs from B6 mice were co-cultured with P29mtB6 or P29mtSAMP1 cybrids; the supernatant was harvested to measure cytokine production. We assessed whether IL-6, TNF-α, MCP-1, IL-10, or IL-12p70 secreted from DCs possibly activated NK cells. IL-6 secretion was increased exclusively after the co-culture of DCs with P29mtSAMP1 cybrids, whereas no significant differences between P29mtSAMP1 and P29mtB6 cybrids were detectable in other cytokines produced by DCs, such as TNF-α, MCP-1, IL-10, and IL-12p70. **P < 0.01.

To obtain insight into how DCs are involved in tumor suppression of P29mtSAMP1 cybrids, we carried out *in vitro* co-cultivation of DCs from B6 mice and P29mtSAMP1 cybrids. We expected that B6-derived DCs somehow discriminate P29mtSAMP1 cybrids from P29mtSAMP1, because DCs are central to trigger the innate immune responses. We then compared the amounts of cytokines produced by DCs, and found that IL-6 secretion was exclusively increased after the co-cultivation of DCs with P29mtSAMP1 cybrids (Figure 5). This suggests that P29mtSAMP1 cybrids are more pro-inflammatory in the host B6 environment than are P29mtB6 cybrids.

### Mechanistic evaluation of immune surveillance against P29mtSAMP1 cybrids

We next investigated how alterations of mtDNA in P29mtSAMP1 cybrids elicited the innate immune response dependent on NK cells and DCs. Comparison of registered mtDNA sequences of B6 mice and SAMP1 mice [17] revealed that only four mutations are present in SAMP1 mtDNA (Table S2): a point mutation in the 16S *rRNA* gene, a silent mutation in the *ND3* (NADH dehydrogenase subunit 3) gene, a missense mutation in the *ND4* gene, and a silent mutation in the *ND5* gene. In addition, we newly identified one additional missense mutation in the *ND5* gene by determining the entire mtDNA sequence of P29mtSAMP1 cybrids (Table 1).

**Figure 6:**
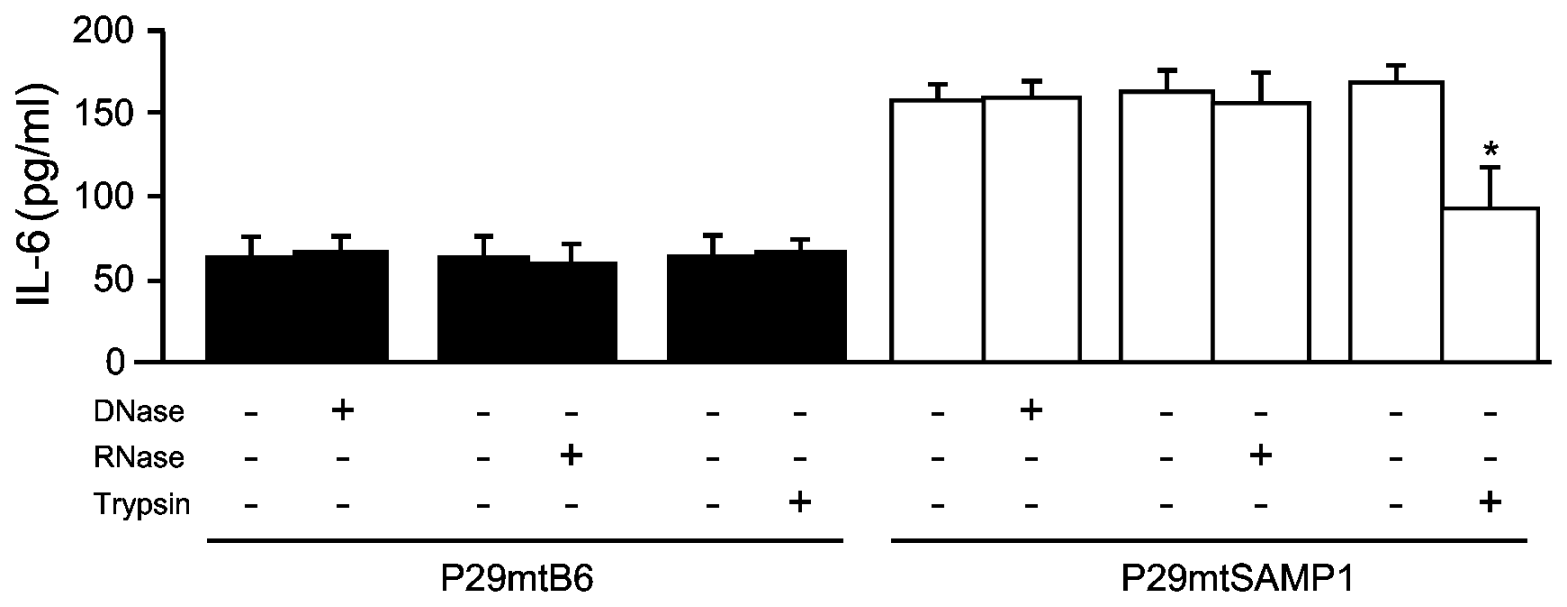
Identification of mitochondrial components that are recognized by DCs. We assessed the effect of pretreating P29mtSAMP1 cybrids with DNase, RNase, or trypsin on the secretion of IL-6 from DCs because not only mtDNA-encoded peptides but also mtDNA itself and the mitochondrial RNAs transcribed from mtDNA could be mitochondrial components that are specifically recognized by DCs and that result in enhanced secretion of IL-6. *P < 0.05.

In light of these observations, we sought to determine which components in P29mtSAMP1 cybrids, i.e. the SAMP1 mtDNA itself, its-related transcripts, or peptides/proteins, define IL-6-inducible, pro-inflammatory nature of P29mtSAMP1 cybrids. To this end, we pretreated P29mtSAMP1 cybrids either with DNase, RNase, or trypsin, and then co-cultivated them with B6 DCs to test the effects of the pretreatment on the secretion of IL-6 from DCs. Whereas DNase or RNase pretreatment had no effect on the amount of IL-6 secreted from DCs, trypsin pretreatment significantly inhibited IL-6 secretion (Figure 6). These results suggest that peptides/proteins probably expressed on cell surface of P29mtSAMP1 cybrids, but not any nucleic acid components including mtDNA itself or transcripts of SAMP1 mtDNA, possess a potential to modify DC functions to regulate the innate immune response.

**Figure 7:**
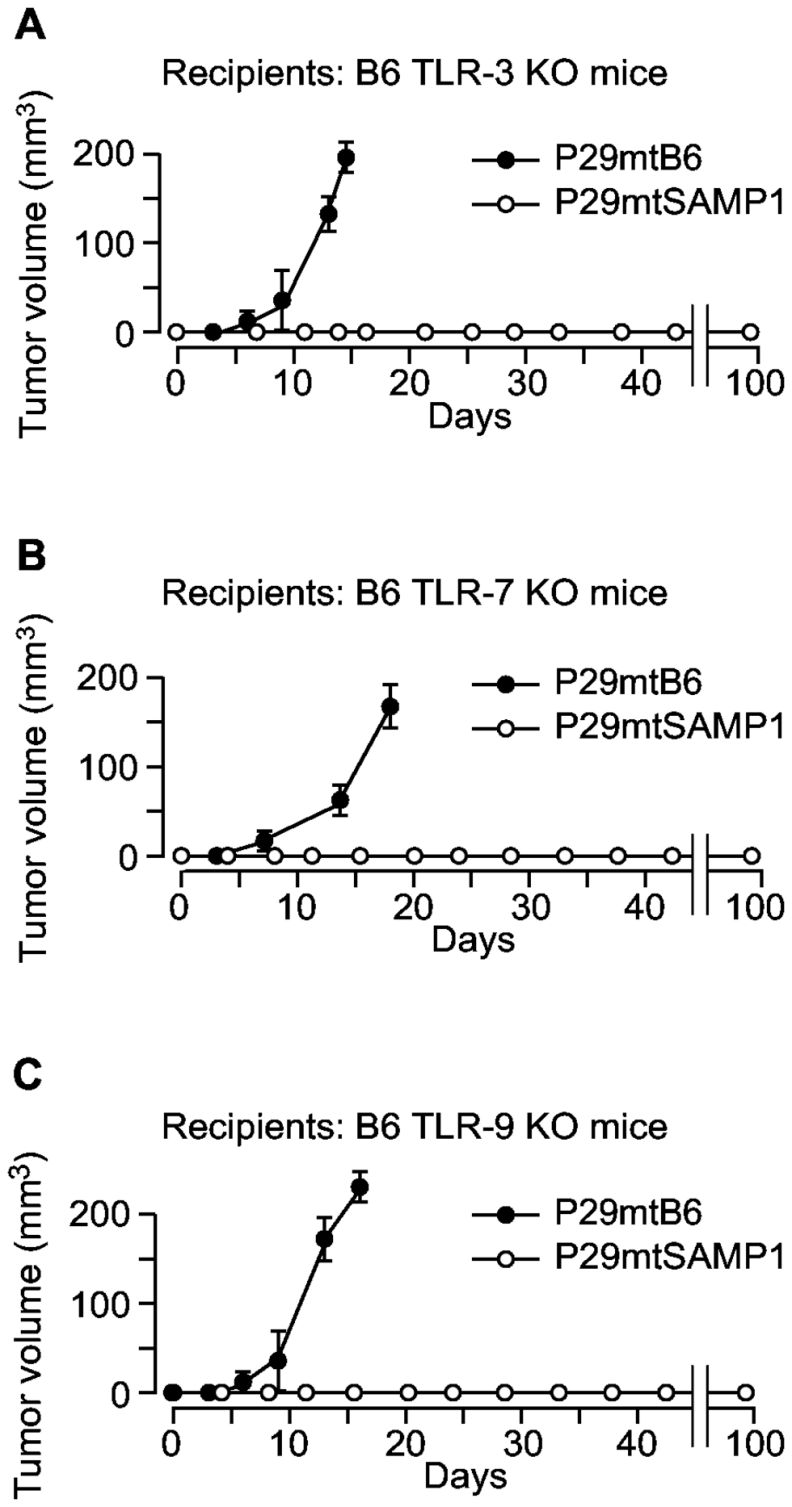
Effects of the absence of Toll-like receptors in B6 mice on tumor formation of P29mtSAMP1 cybrids. To confirm the idea that mtDNA and its transcripts are not the mitochondrial components in P29mtSAMP1 cybrids that are recognized by innate immune cells in B6 mice, we assessed tumor formation of P29mtSAMP1 cybrids in B6 mice that lacked (**A**) TLR-3 (**B**) TLR-7, or (**C**) TLR-9, which recognized double-stranded RNA, single-stranded RNA, and double-stranded DNA, respectively.

We further examined tumor formation of P29mtSAMP1 cybrids in B6 mice without nucleic acid-recognizable TLRs to carefully make sure whether some nucleic acid components-dependent elicitation of immune responses could be involved in the tumor suppression phenotype of P29mtSAMP1 cybrids. Although all TLRs are pattern recognition receptor, and none of examined TLRs is able to distinguish mtDNA sequence with or without mutations, it is also possible that changes of the conformation or changes of the localization of mtDNA fragments as the results of the mtDNA mutations may be recognized by pattern recognition receptors. The B6 mice deficient in TLR-3, 7, or 9, which recognizes double-stranded RNA, single-stranded RNA, and double-stranded DNA, respectively, still maintained an ability to suppress tumor formation of P29mtSAMP1 cybrids (Figure 7). Thus, these results exclude a possibility that nucleic acid components including SAMP1 mtDNA directly elicit the innate immune responses through TLRs.

## Discussion

Our previous study [8] showed that P29mtA11 cybrids with mtDNA from highly metastatic Lewis lung carcinoma cells (A11 cells) expressed ROS overproduction, which induced tumor progression (metastasis). In the current study, we transferred mtDNA from SAMP1 mice into ρ^0^ P29 cells, isolated new transmitochondrial P29mtSAMP1 cybrids that overproduce ROS, and examined whether mtDNA mutations that induced ROS overproduction always confer tumor progression. The results unexpectedly showed that some of the mutations in mtDNA from SAMP1 mice conversely suppressed tumor phenotypes (Figure 1A) due to activation of innate immune responses mediated by DCs and NK cells (Figures 1–3). This finding suggests that some mtDNA mutations in mice function as tumor suppressors (Figure 1), while the other mtDNA mutations function as tumor enhancers [8], even though they both induce ROS overproduction.

It may be possible that the mtDNA mutations in P29mtSAMP1 cybrids cause ROS-mediated protein denaturation, and the denatured proteins consequently enhance antigenicity of P29mtSAMP1 cybrids. However, our previous study [16] reported that P29mtNZB cybrids possessing mtDNA from NZB mice did not show ROS overproduction, but showed suppression of tumor formation in host B6 mice through elicitation of the innate immune responses in the absence of ROS overproduction. Moreover, P29mtA11 cybrids showed significant ROS overproduction, but showed enhanced tumor formation in host B6 mice [8]. These observations suggest that ROS-mediated protein denaturation would not be involved in activation of innate immune cells, although we could not completely exclude this possibility. Probably, alterations in structure or expression levels of proteins and/or nucleic acids arose from mtDNA mutations in some way trigger activation of the innate immune cells.

In the case of P29mtNZB cybrids [16], mtDNA from NZB strain mice harbored more than 90 mutation sites (Table S2). Therefore, it was difficult to determine which mutations are responsible for the recognition by the innate immune system. In contrast, the mtDNA of P29mtSAMP1 cybrids possessed only five mutations compared with the mtDNA of B6 mice (Table 1). Moreover, pretreatment of P29mtSAMP1 cybrids with RNase, DNase, and protease, respectively (Figure 6) revealed that some peptides that arose from P29mtSAMP1 cybrids, but not mtDNA fragments or transcripts of mtDNA, are candidate mitochondrial components recognized by the innate immune cells. We confirmed that B6 mice deficient in pattern recognition receptors of nucleic acids still maintained an ability to suppress tumor formation of P29mtSAMP1 cybrids, suggesting that changes of the conformation or changes of the localization of mtDNA fragments as the results of the mtDNA mutations are not involved in the innate immune recognition.

Enzyme pretreatment experiments of P29mtSAMP1 cybrids revealed that some peptides expressed on P29mtSAMP1 cybrids induce increased IL-6 production by B6 DCs (Figure 6). Although we do not exclude a possibility that other cytokines and/or inflammatory mediators also induced in DCs upon co-cultivation with P29mtSAMP1 cybrids, our results proposed an interesting possibility that peptides might be expressed on the surface of P29mtSAMP1 cybrids, and mediate augmented inflammatory responses around the tumor-inoculated environment. We currently speculate that mtDNA mutations lead to alterations of structures of protein products translated from the transcripts of mtDNA, and that such structural instability or fragility of cellular components may cause stress response to make cells to express stress/danger signals or unusual trafficking of mutated mtDNA-derived components to the cell surface.

Our current results prompt the question of whether any cybrids with mtDNA from allogenic strains (strains other than B6) are recognized by the immune system of host B6 strain mice. Our previous study [16] showed that P29mtC3H cybrids with nuclear DNA from B6-derived P29 cells and mtDNA from C3H strain mice were able to form tumors in B6 mice. Comparison of the full-length sequences of mtDNA from B6 mice and P29mtC3H showed that mtDNA from P29mtC3H cybrids carries only three missense mutations, all of which are different from those in the mtDNA from P29mtSAMP1 (Table 1). This result suggests that missense mutations in mtDNA from different strains are not always recognized by the immune systems of B6 strain mice.

Induced-pluripotent stem (iPS) cells that have the same nuclear DNA background as their nuclear donors are considered to be promising cellular systems for regenerating tissues that will not be rejected after differentiation and transplantation into a host [18–20]. However, our observations suggest that some somatic mutations of mtDNA in tissues could be targets of innate immune cells. Therefore, as the mtDNA in somatic cells accumulates somatic mutations during the aging of the donor, the regenerated tissues from iPS cells with particular somatic mutations in mtDNA could be targets of host innate immune cells, resulting in the rejection of the transplanted tissues.

Many questions remain regarding the mechanism by which innate immune cells, such as DCs and NK cells, selectively recognize and exclude P29mtSAMP1 cybrids. It is also unknown how the ND4 and ND5 peptides, which are translated from the *ND4* and *ND5* genes and which usually integrate into the mitochondrial inner membranes, localize on the cell surface of P29mtSAMP1 cybrids to function as mitochondrial components to be recognized by innate immune cells. In addition, it is unclear which molecules in the innate immune cells recognize the mitochondrial components. Moreover, we could not complete exclude the possibility that ROS overproduction is involved in the specific killing of P29mtSAMP1 cybrids by the innate immune system. Although the precise mechanism needs to be further investigated, our current study provides information that potentially is important for the fields of mitochondrial tumor immunology and regenerative medicine using iPS cells.

## Supporting Information

Table S1 **Genetic characteristics of parent cells and P29mtSAMP1 cybrids.** ^a)^ Fusion mixture was grown in a selection medium without uridine and pyruvate (UP-) that allows exclusive growth of the mtDNA-repopulated ρ^0^P29 cells corresponding to P29mtSAMP1 cybrids. Platelets without nuclei cannot survive, and unfused ρ^0^P29 cells failed to grow in the selection medium due to significant respiration defects caused by the absence of mtDNA.
(XLS)

Table S2 **Comparison of sequences of mtDNA from different mouse strains.** The GenBank accession numbers for the mtDNA sequences of B6, SAMP1, C3H, and NZB are AY172335, AB042524, AB049357, and L07095, respectively. n.p.: nucleic position. a.a. change: amino acid change.
(XLS)
